# High-Resolution Measurement of Local Activation Time Differences From Bipolar Electrogram Amplitude

**DOI:** 10.3389/fphys.2021.653645

**Published:** 2021-04-22

**Authors:** Stephen Gaeta, Tristram D. Bahnson, Craig Henriquez

**Affiliations:** ^1^Inova Heart and Vascular Institute, Falls Church, VA, United States; ^2^Division of Cardiology, Duke University Medical Center, Durham, NC, United States; ^3^Department of Biomedical Engineering, Duke University, Durham, NC, United States

**Keywords:** cardiac electrophysiology, conduction velocity, electrogram, atrial fibrillation, electroanatomic mapping

## Abstract

Localized changes in myocardial conduction velocity (CV) are pro-arrhythmic, but high-resolution mapping of local CV is not yet possible during clinical electrophysiology procedures. This is in part because measurement of local CV at small spatial scales (1 mm) requires accurate annotation of local activation time (LAT) differences with very high temporal resolution (≤1 ms), beyond that of standard clinical methods. We sought to develop a method for high-resolution measurement of LAT differences and validate against existing techniques. First, we use a simplified theoretical model to identify a quantitative relationship between the LAT difference of a pair of electrodes and the peak amplitude of the bipolar EGM measured between them. This allows LAT differences to be calculated from bipolar EGM peak amplitude, by a novel “Determination of EGM Latencies by Transformation of Amplitude” (DELTA) method. Next, we use simulated EGMs from a computational model to validate this method. With 1 kHz sampling, LAT differences less than 4 ms were more accurately measured with DELTA than by standard LAT annotation (mean error 3.8% vs. 22.9%). In a 1-dimensional and a 2-dimension model, CV calculations were more accurate using LAT differences found by the DELTA method than by standard LAT annotation (by unipolar dV/dt timing). DELTA-derived LAT differences were more accurate than standard LAT annotation in simulated complex fractionated EGMs from a model incorporating fibrosis. Finally, we validated the DELTA method *in vivo* using 18,740 bipolar EGMs recorded from the left atrium of 10 atrial fibrillation patients undergoing catheter ablation. Using clinical EGMs, there was agreement in LAT differences found by DELTA, standard LAT annotation, and unipolar waveform cross-correlation. These results demonstrate an underlying relationship between a bipolar EGM’s peak amplitude and the activation time difference between its two electrodes. Our computational modeling and clinical results suggest this relationship can be leveraged clinically to improve measurement accuracy for small LAT differences, which may improve CV measurement at small spatial scales.

## Introduction

Cardiac arrhythmia results from a complex interplay of fixed anatomical and dynamic functional sources that are often times difficult to characterize clinically. Fundamental electrodynamic (King et al., 2013) and clinical studies (Brunckhorst, 2002; Jaïs et al., 2012; Nayyar et al., 2014; Irie et al., 2015; Jackson et al., 2015; Anter et al., 2016, 2018a,b) have suggested that areas of slow conduction velocity (CV) are likely to be pro-arrhythmic. There are multiple methods to calculate CV (Cantwell et al., 2015; Masse et al., 2016). Most commonly, CV is calculated using the local activation time (LAT) differences and spatial separation of sets of recorded EGMs (the time to cover a known distance defines speed). Given three EGMs, a local CV vector describing the speed and direction of wavefront propagation can be “triangulated” in this way (Dubois et al., 2012; Cantwell et al., 2015; Verma et al., 2016, 2018; Zheng et al., 2017; Anter et al.,2018a,b). The accuracy of this method is necessarily dependent on the spatial accuracy of distance measurements and the temporal accuracy of LAT differences.

Conduction velocity can theoretically be calculated from any pair of electrodes given known inter-electrode distance and the difference in their activation times. However, the temporal resolution of LAT annotation using standard clinical systems with 1 kHz sampled data is 1 ms, which is insuficient for measurement of timing differences between very closely spaced electrodes. By way of example, if CV is 80cm/sec (0.8 mm/ms), the LAT difference for a 1 mm separated electrode pair would be 1.25 ms, but the system cannot accurately resolve below 1 ms. We therefore sought to develop a technique to measure small LAT differences to enable accurate CV calculation from closely spaced electrodes with available clinical systems.

Previous work identified a close relationship between the peak (Gaeta et al., 2020) or peak-to-peak (Mendonca Costa et al., 2020) amplitude of a bipolar EGM and the LAT difference between its component electrodes. We hypothesized that LAT difference between an electrode pair could therefore be calculated from their resulting peak bipolar amplitude [hereafter the Determination of Electrogram Latencies by Transformation of Amplitude (DELTA) method]. Measurement of these timing differences in the more finely sampled voltage domain could allow resolution of LAT differences below the 1 ms data sampling rate. We use a simplified theoretical model of a bipolar EGM to derive the DELTA method, validate its predictions using EGMs from both clinical recordings and 2D models of healthy and fibrotic cardiac tissue, and demonstrate its ability to enhance the accuracy of CV measurement between closely spaced electrodes.

## Materials and Methods

### Computational Model

To obtain a ground truth for the inter-electrode LAT, we used a 2D computational model of cardiac tissue. In this model, the transmembrane voltage is available to measure wavefront arrival times, offering a ground truth for inter-electrode LAT differences. Additionally, tunable tissue conductivity allows changes in CV to be assessed and to generate random, microholes as a representation of fibrosis (Gokhale et al., 2018). A 2cm x 2cm sheet of tissue was simulated using the Courtemanche et al. human atrial cell model. Simulated electrograms were calculated during a planar wave of excitation at pairs or triads of sites (“simulated electrodes”). Extracellular potentials (*U*) at these sites were output at a precision of 0.001 mV and sampling frequency of 200 kHz. See Supplementary Material for model details.

### LAT Annotation by Maximum Negative dV/dt

The LAT of each simulated and clinical unipolar EGM was annotated as the timing of its steepest unipolar EGM slope (maximum negative *dV/dt*) within a window of 20 to 300 ms after stimulation (to avoid stimulus artifact and repolarization).

### Amplitude Normalization

Prior to DELTA analysis, unipolar EGM pairs were amplitude normalized before calculation of their resulting bipolar EGM. Peak to peak unipolar amplitude (*U*) of each signal was measured in a 30 ms window surrounding its maximum negative *dV/dt*. Each unipolar EGM was then scaled to 1 mV peak to peak amplitude, centered on 0 mV. The baseline percentage difference in paired unipolar amplitudes was calculated prior to normalization as the absolute value of their peak to peak amplitude difference divided by their mean.

### DELTA Method

To calculate LAT differences using the DELTA method, bipolar EGMs were calculated as the difference between pairs of amplitude normalized unipolar signals (as above). Using Eq. 2, the LAT difference (*τ*) is calculated from the peak voltage amplitude of this resulting bipolar EGM (*B*), the unipolar peak to peak amplitude (*U*, normalized to 1 mV), and the mean maximum negative *dV/dt* of the two normalized unipolar signals (*m*).

### Patient Study

10 patients with a history of AF (5 persistent, 5 paroxysmal) undergoing a planned ablation procedure were enrolled. Following documented pulmonary vein isolation (PVI), a high density map of the left atrial posterior wall (LAPW) was created during coronary sinus pacing at 600 ms cycle length using a 2-6-2 PentaRay multipolar catheter and the Carto3 mapping system (Biosense Webster, Diamond Bar, CA, United States). Unipolar electrograms were recorded relative to Wilson’s central terminus and bandpass filtered at 2-240 Hz prior to export. Signals were sampled at the nominal rate of 1 kHz with 0.003 mV voltage steps. This study was approved by the Duke University Medical Center Institutional Review Board and all subjects granted informed consent prior to enrollment. See Supplementary Material for additional study and data collection details.

### Electrode Pair Definition

The PentaRay multipolar mapping catheter has inter-electrode spacing of 2-6-2 mm, yielding two bipolar pairs with approximately 2 mm center-to-center (1 mm edge-to-edge) electrode distance on each of five splines. We defined additional bipolar pairs consisting of all possible electrode pairs on a given PentaRay spline. In this way, each of the five splines yielded two standard 2 mm bipoles in addition to a 6 mm, two 8 mm, and one 10 mm electrode pair. Bipolar pairs spanning splines were not created in order to ensure fixed inter-electrode distance and avoid spatial inaccuracy in inter-electrode distance measurements. For each electrode pair a bipolar electrogram was calculated by subtracting the more proximal unipolar electrogram from the more distal electrogram of the pair.

## Results

### Theoretical Model of a Bipolar EGM

We hypothesized that the peak voltage amplitude of a bipolar EGM quantitatively encodes the LAT difference between its component unipolar signals. To derive this relationship, we created a simplified theoretical model of a bipolar EGM as the difference between two unipolar waveforms. To reduce complexity our model considers only the unipolar downstroke, which we approximate as sinusoidal. Its accuracy is therefore limited to electrode pairs with small LAT differences, in which both unipolar downstrokes occur nearly simultaneously. We note that this is the most near- field period, during which the spreading wave of excitation passes beneath a pair of recording electrodes (Spach et al., 1972). A full derivation of the theoretical model is found in the Supplementary Material. We modeled the unipolar electrogram as a traveling sinusoidal wave on a 1-dimensional cable, and a pair of unipolar electrograms as simultaneous measurements at sites separated by the inter- electrode distance (*d*). In this model, the bipolar EGM is the difference between two equivalent sinusoids and its peak amplitude will therefore vary as a function of the unipolar phase difference. This model predicts that for a pair of unipolar electrograms with equal peak to peak amplitude, *U*, the peak bipolar amplitude (*B*) will be a function of their amplitude and phase difference (*φ*), as:

(1) $$B=U\,\sin\frac{\phi }{2}$$

The LAT difference (*τ*) between an electrode pair can be calculated from its peak bipolar voltage amplitude by:

(2) $$\tau =\frac{U}{m}\,\sin^{-1}\frac{B}{U},$$

where *m* is the most negative slope of the unipolar signals (maximum negative *dV/dt*).

For unipolar signals with equivalent downstrokes (equal amplitude and frequency), the peak amplitude of the resulting bipolar electrogram encodes the unipolar LAT difference by Eq. 2. As described in the Supplementary Material, the peak of the bipolar waveform will always occur during the period of simultaneous downstrokes (when model assumptions are valid). Importantly, we also show that this relationship holds regardless of the ordering of unipolar electrodes relative to the wavefront or, equivalently, whether the bipolar peak is positive or negative.

### Clinical EGM Database

We sought to test our prediction that inter-electrode LAT differences can be measured by the DELTA method *in vivo* using clinically recorded intracardiac EGMs. To do so, we aimed to create a database of bipolar EGMs with varying LAT differences. We hypothesized that even during planar wave propagation, variations in the angle of incidence between a wavefront of activation and bipolar electrode pairs would provide a natural catalog of time delays with which to validate these calculations.

EGMs were recorded from the LAPW of 10 AF patients following pulmonary vein isolation. Coronary sinus pacing resulted in an approximately planar wave of excitation ascending the LAPW. A total of 14,168 unipolar electrograms (7,084 standard bipoles) were recorded using the 20-pole catheter, with a mean 708.4 per patient (range 456–1180). Noting that LAT differences will vary by inter-electrode spacing, we analyzed electrode pairs with inter-electrode spacings of 2, 6, 8, and 10 mm center-to-center. In this way, the initial set 7084 bipolar electrograms (2 mm spacing) yielded a database of 18,740 bipolar electrograms with variable inter-electrode spacing.

### Clinical EGM Characteristics

The mean recorded unipolar peak to peak amplitude was 1.76 *±* 0.34 (0.044 to 7.73) mV. The measured maximum negative *dV/dt* for each unipolar electrogram was strongly correlated with its peak to peak amplitude, with an apparent linear relationship (Supplementary Figure 3B). 2 mm electrode pairs had unipolar peak to peak amplitudes differing by a mean of 19.1% (mean 42.9 *±* 37.6% for all pairs; Supplementary Figure 3C). Following amplitude normalization, the maximum negative *dV/dt* for each unipolar electrogram was more tightly constrained, with a mean of −0.17 *±* 0.059 (−0.005 to −0.56) mV/ms (Supplementary Figure 4).

### Unipolar Amplitude Normalization

Although the model predicts that peak bipolar voltage amplitude will encode unipolar LAT difference, its assumption of equivalent unipolar downstrokes (with equal amplitude and frequency) is clearly only an approximation when analyzing real clinical data. In reality, the waveforms of adjacent unipolar EGMs often differ even with closely spaced electrode pairs. Prior modeling work suggests a major contributor to adjacent unipolar differences is oblique catheter orientation relative to the tissue (Schuler et al., 2013). Progressive catheter inclination leads one unipolar electrogram to have smaller amplitude and lower frequency (a more “far-field” signal). This violation of the theoretical model’s assumptions will impact the accuracy of Eq. 2 in predicting LAT differences from bipolar amplitude.

**FIGURE 1:**
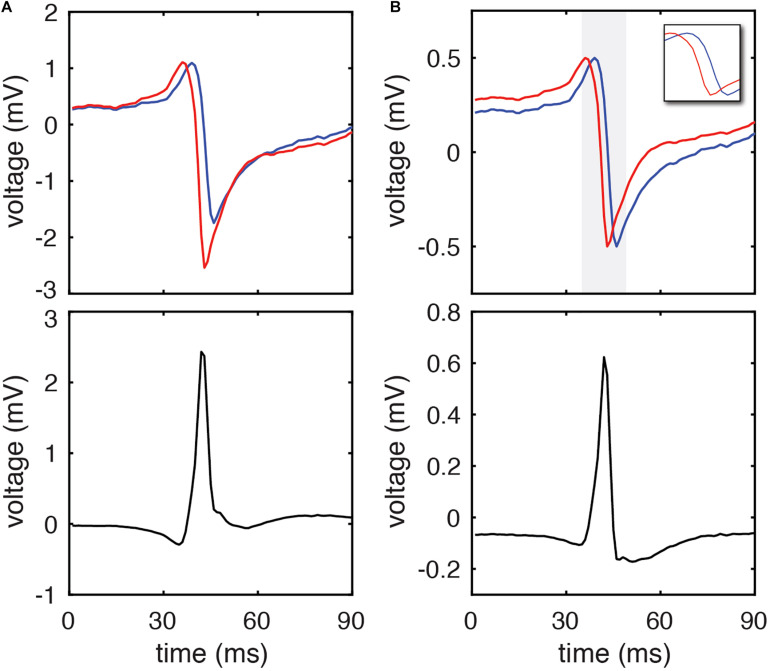
DELTA method calculation of inter-electrode LAT difference using clinical EGMs. **(A)** A representative pair of unipolar electrograms recorded from 2 mm center-to-center spaced electrodes (blue and red) and their calculated bipolar electrogram (black). Using the timing of their maximum negative *dV/dt* (the standard activation time annotation method) their activation time difference is 2 ms. **(B)** The same unipolar electrograms (blue and red) after amplitude normalization to a range of 0 to 1 mV. The period of the unipolar downstrokes (gray) is isolated in the inset. The peak amplitude of the resulting bipolar electrogram (black) occurs during the phase of simultaneous unipolar downstrokes, and by DELTA predicts a –2.191 ms time delay between the two unipolar signals.

**FIGURE 2:**
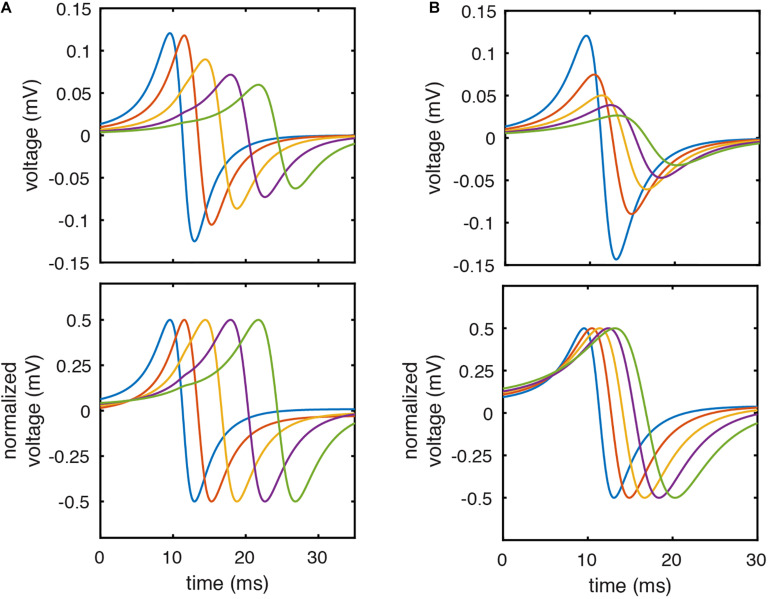
Unipolar amplitude normalization corrects for differences in EGM morphology due to electrode-tissue distance or CV. **(A)** Simulated unipolar EGMs with electrode- tissue distances of 1.5, 2.25, 3.25, 4.25, and 5.25 mm before (top) and after (bottom) amplitude normalization. **(B)** Simulated unipolar electrograms from model locations with CV of 70.0, 62.9, 56.0, 50.9, and 46.8 cm/sec before (top) and after (bottom) amplitude normalization.

We hypothesized that this limitation could be (partially) corrected by normalizing the peak to peak amplitude of each pair of unipolar EGMs prior to calculating their resulting bipolar waveform. This approach is similar to that utilized by prior studies of bipolar EGM morphology (Mendonca Costa et al., 2020). The effect of this approach on the morphology of clinically recorded EGMs is seen in Figure 1. Normalization leads to identical unipolar amplitudes and correction of differences in downstroke slope. Using simulated EGMs, amplitude normalization similarly corrects (imperfectly) for unipolar morphology differences due to differing electrode-tissue distance (Figure 2A) or differing CV (Figure 2B).

**FIGURE 3:**
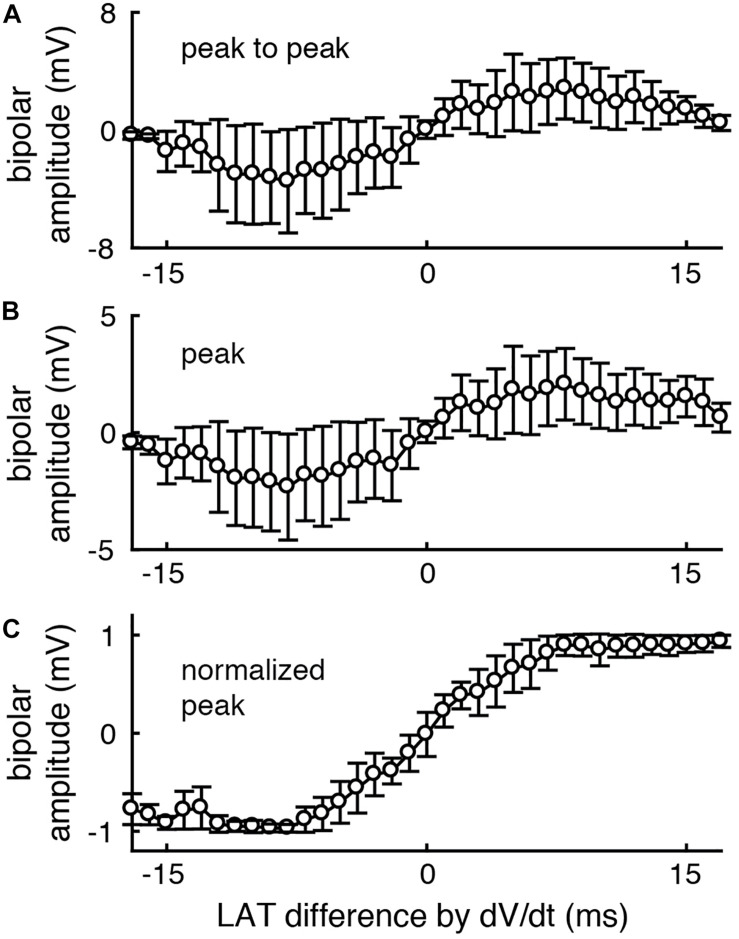
After unipolar amplitude normalization, peak bipolar voltage amplitude more closely encodes inter-electrode LAT differences. The peak to peak **(A)** and peak **(B)** voltage amplitude of each clinically recorded bipolar EGM is correlated with the LAT difference between its component unipolar EGMs. **(C)** After normalizing unipolar EGMs to 1 mV amplitude (peak to peak), the peak voltage of the resulting bipolar EGMs more closely encodes the unipolar LAT differences (bottom). *n* = 18740. Error bars represent the standard deviation.

We next tested the effect of unipolar amplitude normalization on bipolar voltage amplitude using our database of clinically recorded electrograms (*n* = 18,740). As expected, the peak to peak (top) and peak (middle) bipolar amplitude of clinically recorded electrograms increases as the inter- electrode LAT difference increases (here calculated by the standard method (timing of maximum negative unipolar *dV/dt*)). When bipolar EGMs are calculated following amplitude normalization of unipolar EGMs, there is much less variation in peak bipolar amplitude for a given LAT difference (Figure 3).

### LAT Difference Measurement Accuracy

**FIGURE 4:**
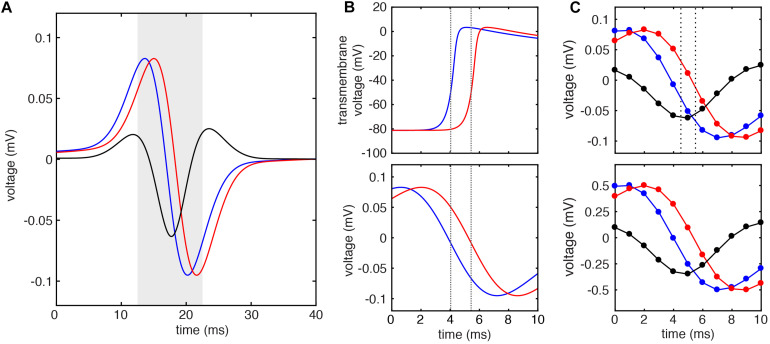
Example LAT difference calculation by standard and DELTA methods using simulated EGMs. **(A)** Unipolar electrograms (*U*) and calculated bipolar electrogram (*B*) recorded from simulated electrodes with inter-electrode distance of 1 mm. **(B)** Transmembrane voltage (*V*) of simulated cells at the site of unipolar electrograms in **(A)** (top). Local activation times calculated by threshold crossing of the action potential upstroke (dashed lines) reveal ground truth inter-electrode time delay of 1.400 ms. This same inter-electrode time delay is found by the standard method (maximum negative *dV/dt* timing) using the 200 kHz sampled unipolar electrograms (bottom). **(C)** When these unipolar signals are sampled at 1 kHz, the inter-electrode time delay calculated by the standard method is 1 ms (dashed lines, top). If instead, the inter-electrode time delay is calculated by DELTA using the amplitude normalized signals (bottom), an inter-electrode time delay of 1.404 ms is found.

To test the above predictions, we used model data to compare the LAT differences predicted by Eq. 2 against both the ground truth LAT differences (measured by the timing of action potential upstroke) and LAT differences measured by the standard method (by the timing of maximum negative unipolar *dV/dt*). Figure 4 shows an example calculation for a simulated electrode pair separated by 1 mm and 1.5 mm above the tissue. Note that the downstrokes of these unipolar signals have approximately sinusoidal morphology (Figure 4B, bottom). When the LATs at these sites are calculated by the timing of action potential upstroke in the simulated cell beneath each simulated electrode (Figure 4B, dashed lines), a ground truth LAT difference of 1.400 ms is found. Note that simulated EGM sampling frequency of 200 kHz allows temporal resolution of 0.005 ms.

To simulate clinical data, the extracellular potentials were next downsampled to 1 kHz (Figure 4C, top), allowing temporal resolution of only 1 ms. Using these signals, the LAT difference found by the timing of unipolar maximum negative *dV/dt* is 1 ms (dashed lines). To calculate the LAT difference by the DELTA method, these (downsampled) unipolar signals were normalized to a peak-to-peak amplitude of 1 mV, after which a new bipolar EGM was calculated as their difference (Figure 4C, bottom). Given the measured unipolar maximum negative *dV/dt* of -0.254 mV/ms (after normalization) and peak bipolar amplitude of -0.349 mV, the LAT difference predicted by the DELTA method (Eq. 2) is 1.404 ms, nearly identical to the ground truth. Note that by using measurements in the more finely discretized voltage domain (with voltage step 0.001 mV), a higher temporal resolution is afforded by this method compared to the standard, time-domain method.

**FIGURE 5:**
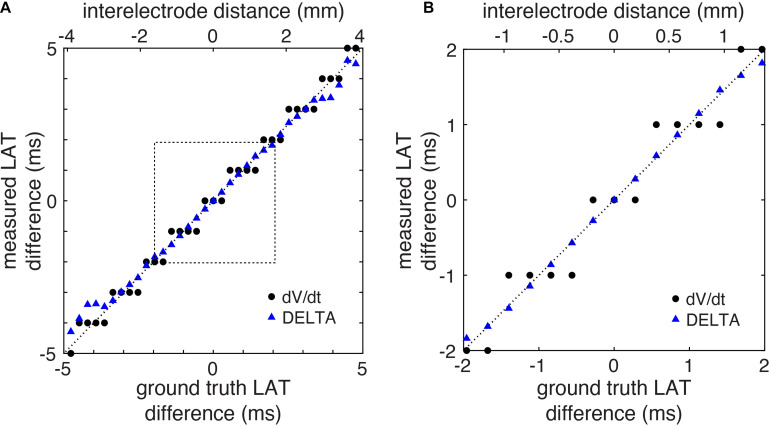
LAT differences measured by DELTA are accurate in a computational model. **(A)** LAT differences measured by DELTA (triangles) more closely reproduce ground truth measurements (dashed line) compared to standard technique (circles) in the computational model. **(B)** Focusing on LAT differences of 2 ms or less (corresponding to dashed box in Panel A) it is seen that in spite of using the same 1 ms sampled EGMs, by using the higher resolution voltage data the DELTA method avoids the 1 ms discretization of measured LAT differences measurements produced by the standard LAT annotation technique.

Using this 2D model, we systematically studied the accuracy of LAT difference measurements by DELTA compared to ground truth and to the standard technique (maximum negative dV/dt). A total of 41 electrode pairs were simulated with inter-electrode distances from 0 to 4 mm. For this study, electrode pairs were oriented parallel to the propagating wavefront. This led to ground truth LAT differences from about −6 to +6 ms. As in the example above, for DELTA and dV/dt measurements, EGMs were filtered and downsampled to 1 kHz to simulate clinically realistic data. For each electrode pair, the ground truth LAT difference (found using 200 kHz sampled Vm data) was compared to LAT difference measured by maximum negative dV/dt annotation and by DELTA. As seen in Figure 5, the DELTA method (triangles) accurately measured the ground truth LAT difference (dashed line) for LAT difference less than approximately ±4 ms. This is in agreement with the theoretical prediction of accuracy only at LAT differences small enough for unipolar downstrokes to overlap. Despite using 1 ms sampled EGMs, the DELTA method avoided the 1 ms discretized results inherent to the standard, time-based annotation technique. Overall, for LAT differences less than 4 ms (*n* = 29), DELTA-derived LAT differences were more accurate than standard technique, with mean error of 0.095 ms (3.77%) compared to 0.245 ms (22.86%), respectively.

### Fibrosis Model

To explore the performance of the DELTA method on more complex EGMs, additional 2D simulations were performed in which fibrosis was incorporated by creating random microholes throughout the domain to generate discontinuous wavefront propagation. On these more complex EGMs (Supplementary Figure 6), the DELTA method more closely approximated the ground truth LAT differences than standard LAT annotation. See Supplementary Material for details.

### Local CV Measurement Accuracy – 1D

To demonstrate the benefit of this higher temporal resolution when calculating CV at small spatial scales, we calculated CV in the computational model using LAT differences found by action potential upstroke time (ground truth), the standard method (timing of maximum negative *dV/dt*), and the DELTA method. CV is calculated here by finite difference (*CV* = *d/τ*) using LAT differences (*τ*) between adjacent electrodes separated by inter-electrode distances of 1 mm (*d*). For this experiment, electrodes were oriented parallel to the planar wavefront, in effect measuring CV along 1-dimension. The model had linearly decreasing conductivity, resulting in decreased CV (and increasing inter-electrode LAT differences) along its length.

**FIGURE 6:**
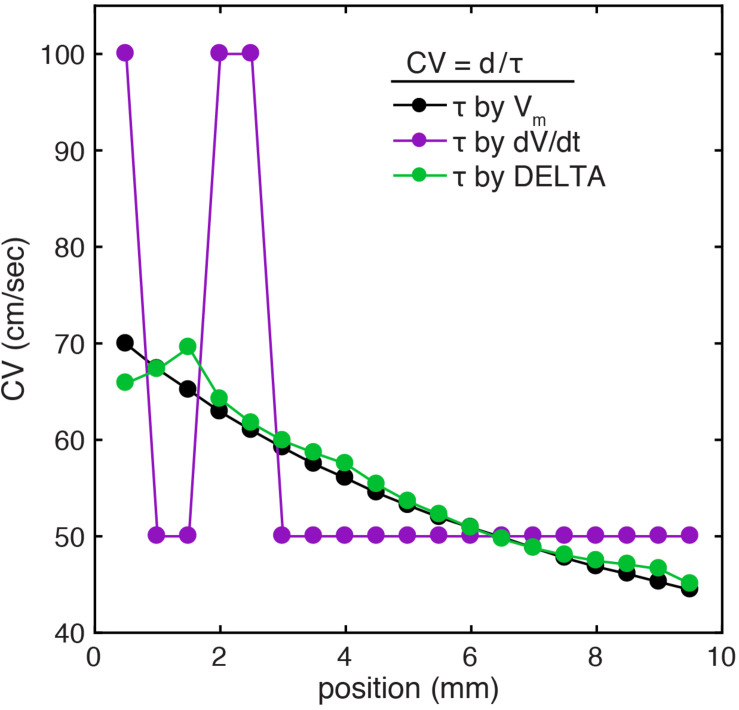
CV calculation using DELTA-derived LAT differences improves accuracy at small spatial scales in a 1D computational model. **(A)** In the setting of linearly decreasing tissue conductivity, CV decreases along the length of the simulated sheet. Results are shown for CV calculation by inter-electrode time delays between 1 mm separated simulated electrodes. When compared to CV calculated using time delays found by action potential upstroke time (green), CV calculated with time delays found by timing of unipolar maximum negative *dV/dt* (purple) are inaccurate due to only 1 ms temporal resolution (resulting in only time delays of 1 or 2 ms in this example). If instead time delays are calculated by the DELTA method (green), the resulting calculated CV closely recreates the ground truth.

As seen in Figure 6, this model has monotonically decreasing CV as measured by the ground truth LAT differences (black). When LAT differences are calculated from the downsampled data (1 kHz) by the standard method, CV measurements are highly inaccurate due to LAT measurements at a coarse temporal resolution of 1 ms (mean error 16.8%, range 0.25–64%). When CV is instead calculated from the downsampled data using LAT differences found by the DELTA method, the higher effective temporal resolution results in more accurate measurement of CV (mean error 1.7%, range 0.02–6.8%). Notably, changing CV along the length of the simulated tissue led to waveform differences within each pair of unipolar electrograms (seen in Figure 2B). The accuracy of the DELTA method supports the ability of amplitude normalization to compensate for unipolar waveform differences in this context.

### Local CV Measurement Accuracy–2D

We hypothesized that higher resolution LAT difference measurement would improve the accuracy of CV calculation by any method dependent on LAT difference annotation. To test this, we calculated CV from triads of electrodes in the 2D model using a previously described triangulation method (Cantwell et al., 2015). By this method, a 2-dimensional CV vector can be triangulated from any triad of electrodes if the LAT difference across each edge is known.

We simulated a triad of electrodes with 2 mm inter-electrode spacing during planar wavefront propagation. The orientation of the triad was rotated by 15 degree increments through 360 degrees and CV was triangulated each time using LAT differences found by the action potential upstroke (ground truth), standard technique (maximum negative dV/dt), and the DELTA method. For both the standard technique and the DELTA method, EGMs were filtered and downsampled to 1 kHz to recreate clinically realistic data.

**FIGURE 7:**
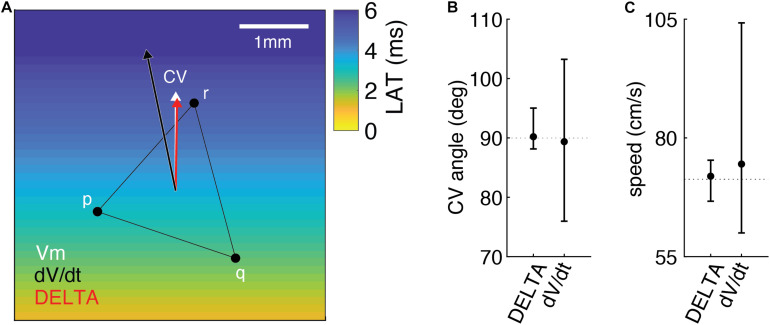
Measuring LAT differences using DELTA improves the accuracy of 2D CV measurement in the computational model compared to standard LAT measurement technique. **(A)** Example of 2D CV triangulation from a triad of electrodes in the model, with planar wavefront propagation from the inferior edge. When compared to the ground truth CV (white arrow), CV calculated using DELTA-derived LAT differences (red arrow) is more accurate than that calculated using LAT differences found by standard technique (black arrow). See text for additional details. CV angles **(B)** and speeds **(C)** calculated from triads rotated by 15 degree increments through 360 degrees (*n* = 25). When compared to ground truth (dashed lines), CV angle and speeds calculated using DELTA-derived LAT differences are more accurate than using standard LAT measurement technique (maximum negative dV/dt timing). Shown are means with error bars representing ranges.

An example of this method is seen in Figure 7A. For this triad of electrodes, the ground truth LAT differences along each edge of the triangle (t_*a*_, t_*b*_, and t_*c*_) are −0.84, 1.965, and 2.805 ms. From these LAT differences, a ground truth CV of 71.3 cm/sec at an angle of 90 degrees (white arrow) can be calculated by a previously described triangulation method (Cantwell et al., 2015). When DELTA-derived LAT differences of −0.849, 2.136, and 2.731 ms are used, a CV of 66.664 cm/sec at an angle of 89.019 degrees is found (red arrow). Finally, using LAT differences measured by standard technique (maximum negative dV/dt) of −1, 1, and 2 ms the calculated CV is 104.213 cm/sec at an angle of 103.241 degrees (black arrow).

The results of CV angle and speed measurements for all triad orientations are seen in Figures 7B,C. CV angle measurements using DELTA derived LAT differences were more accurate than with standard LAT measurement, with mean error 1.386 degrees (range −1.861 to 5.022 degrees) and 7.831 degrees (range −14.036 to 13.241 degrees), respectively. Likewise, speed measurements using DELTA-derived LAT differences were more accurate than with standard LAT measurement, with mean error 0.634 cm/sec (range −4.634 to 4.008 cm/sec) and 3.193 cm/sec (range −11.298 to 32.915 cm/sec), respectively.

### *In vivo* Testing

For each pair of unipolar EGMs, an inter-electrode LAT difference was calculated by the clinical gold standard method (using timing of maximum negative *dV/dt*), waveform cross-correlation (Shors et al., 1996; Fitzgerald et al., 2003), and the DELTA method. A representative clinical EGM pair is shown in Figure 1, before (A) and after (B) normalizing unipolar peak to peak amplitudes to 1 mV. For this set of unipolar EGMs, the maximum negative *dV/dt* occurred at time 91 and 93 ms, respectively, giving a time delay of −2 ms by this standard annotation method. Using the amplitude-normalized signals, the mean maximum negative *dV/dt* for this unipolar pair is −0.307 mV/ms and the peak bipolar amplitude is 0.623 mV. By Eq. 25, the time delay calculated by the DELTA method is therefore −2.191 ms.

**FIGURE 8:**
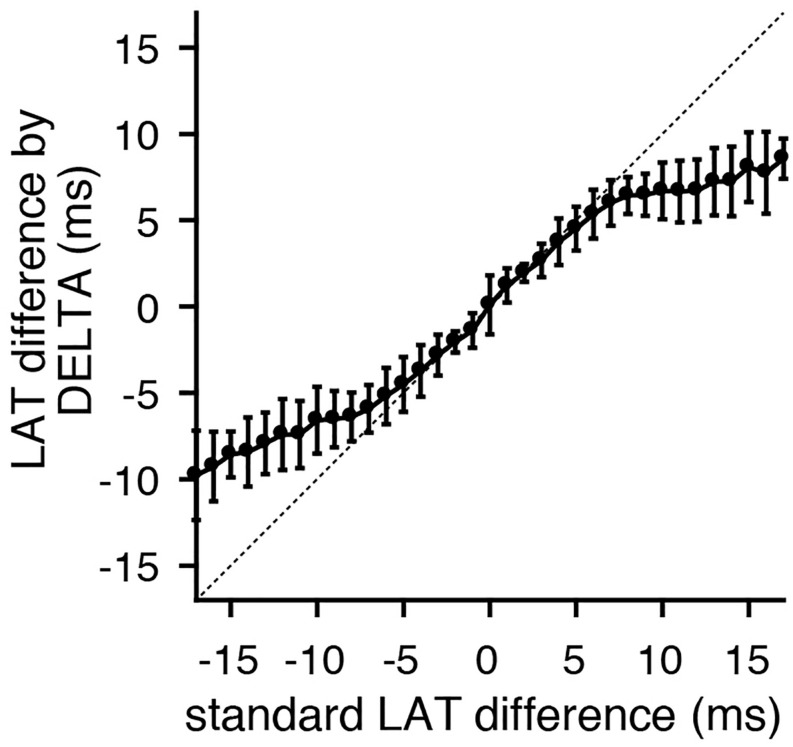
LAT differences calculated by the DELTA method correlate with gold standard time domain measurements. Comparing standard LAT difference measurement (by maximum negative *dV/dt* timing) to LAT differences calculated by the DELTA method reveals a close 1:1 correlation for time delays within approximately ± 5 ms. Shown are the mean (circles) and standard deviation (error bars). Data shown is the collection of all bipole combinations (including all inter-electrode spacings). The identity line (dashed) is included for clarity.

Analyzing the entire data set of clinical electrograms, the LAT differences calculated by the DELTA method have a close, linear correlation with those measured by maximum negative *dV/dt* for time delays less than approximately ± 5 ms (Figure 8). This is in accordance with theoretical predictions that the DELTA will fail with larger LAT differences with non-overlapping unipolar downstrokes. In our data set, this is predicted to occur for LAT differences greater than ± 5.2 ms (the shortest unipolar downstroke duration). Time delays measured by the DELTA method also correlate well with those found by waveform cross-correlation (Supplementary Figure 5).

## Discussion

Recent computational and translational work (Gaeta et al., 2020; Mendonca Costa et al., 2020) has better characterized the relationship between a bipolar EGM’s amplitude and the LAT difference between its component electrodes. Our novel method (DELTA) leverages this relationship to measure small LAT differences between closely spaced electrodes from their resulting peak bipolar voltage amplitude. In addition to validating the theoretically identified relationship, our results suggest a potential application of this method for clinical arrhythmia mapping. By calculating timing differences from voltage data, the high voltage resolution of contemporary EP recording systems can be used to measure small (microseconds) LAT differences that are below the temporal resolution of the systems.

To the best of our knowledge, this is the first study utilizing unipolar amplitude normalization prior to bipolar EGM calculation. Interpretation of clinical bipolar EGMs is challenged by the simultaneous influence of differences in unipolar timing, amplitude, and morphology (Josephson and Anter, 2015). Unipolar signal normalization reduces the influence of amplitude and morphology differences on their resulting bipolar EGM. As seen in Figure 2C, the resulting bipolar EGMs more selectively encode unipolar timing differences. While our approach to unipolar normalization is an imperfect means to reduce the complexity of clinical EGMs, we believe that the utility of reducing bipolar electrogram complexity can outweigh accompanying inaccuracies. Future studies will explore the applicability and limitations of this approach.

The accuracy of the DELTA method is not predicted to be directionally sensitive. As we have previously shown, directional sensitivity of bipolar amplitude is due to changes in LAT difference as an electrode pair is rotated relative to a propagating wavefront (Gaeta et al., 2020). DELTA-derived LAT differences would therefore be expected to be directionally sensitive, but only insofar as LAT differences themselves will vary with changing bipole orientation. Notably, both the clinical data set and the computational model 2D CV study include all catheter orientations. The accuracy of DELTA measurements in the model and the clinical data set in spite of changing bipolar orientations are consistent with this. Likewise, the clinical data set and the 1D model results include electrode pairs with a wide range of inter-electrode distances. This also does not affect the accuracy of DELTA measurements, aside from cases in which the resulting LAT differences are large enough to render the theoretical assumptions invalid (±5 ms in the clinical data set).

The relationship between bipolar voltage amplitude and the underlying unipolar phase difference can be derived from general principles of uniformly propagating waves [see for example (Plank and Hofer, 2018)]. To the best of our knowledge, the present work is the first application of this relationship to determine inter-electrode LAT differences. Noting that a bipolar EGM represents a superposition of two unipolar waveforms (*V*_*B*_=*V*_1_ + (−*V*_2_), Eq. 9), this concept is akin to interferometry techniques in other physical systems, in which wave interference allows measurements of distance or time to be made in the amplitude/intensity domain.

In this proof-of-concept study, the DELTA method improved the accuracy of LAT difference measurement compared to standard LAT annotation. The DELTA method is not a CV calculation technique, but we anticipate that the improved resolution of the DELTA method will improve any CV calculation requiring LAT difference measurement. In the present study, these improvements were seen when CV was calculated in 1 and 2 dimensions from these LAT differences using a finite difference and a triangulation method, respectively. Notably, the latter can be clinically implemented with newer catheters with triads of closely spaced microelectrodes (Leshem et al., 2017; Sulkin et al., 2018), theoretically allowing local CV measurement with each individual point acquisition given sufficient temporal resolution. Importantly, given the scale-dependence of CV, measurement at macroscopic distances will differ from that measured at the smallest scales. The clinical utility of CV measurement over differing scales remains to be explored.

Improved measurement of small LAT differences is needed for calculation of truly “local” CV over millimeter distances. Mapping of local CV with such high spatial and temporal resolution using widely available clinical systems has not previously been possible. We hope that novel techniques such as the DELTA method can help to realize this possibility and improve clinical mapping of arrhythmia substrate.

## Limitations

Our simplified theoretical model and the predictions derived from it are impacted by several assumptions. First, we have modeled the unipolar EGM downstroke as a sinusoid. This choice is deliberate, as it results in a simple relationship between unipolar phase difference and peak bipolar amplitude. Deviations from a true sinusoidal downstroke in clinical unipolar EGMs will lead to inaccuracy of the DELTA method, but our results suggest validity in this approximation.

Recent work suggests differential effects on EGM morphology when conduction delays results from changing intracellular conductivity, sodium channel conductance, or fibrosis (Mendonca Costa et al., 2020). Our proof-of-concept study did not explore the effect of varying underlying mechanisms of conduction slowing on the performance of the proposed DELTA method. It remains to be seen whether the differences in our approach—use of peak (rather than peak to peak) bipolar amplitude, unipolar amplitude normalization, and incorporation of the unipolar down-stroke slope (*m*)—affect this result. Our clinical results support that time delays in healthy tissue or tissue with low levels of fibrosis are well determined by this approach.

The clinical data used for testing in this study was recorded during planar wavefront propagation over relatively simple geometries. The performance of this technique in calculating time delays from more complex clinical signals, including those recorded from thicker tissue, during more complicated propagation patterns, with complex fractionated activity, or with more far-field signal remains to be fully explored. Future work will study the effects of electrode size, sampling frequency, and filtering on the performance of this method. Our initial modeling results with incorporated fibrosis (Supplementary Material) suggest that the DELTA method retains accuracy with more complex EGMs, but this remains to be fully explored including the effect of varying fibrosis patterns and density. We hypothesize that the improved temporal resolution afforded by this approach will allow smaller, more closely spaced electrodes with which these more macro-scale complexities will be lessened.

## Data Availability Statement

The raw data supporting the conclusions of this article will be made available by the authors, without undue reservation.

## Ethics Statement

The studies involving human participants were reviewed and approved by IRB of Duke University. The patients/participants provided their written informed consent to participate in this study.

## Author Contributions

SG: concept, design, data collection and processing, and writing. TB: supervision, resources, data collection, and critical review. CH: design, supervision, resources, analysis and interpretation, and critical review. All authors contributed to the article and approved the submitted version.

## Conflict of Interest

The authors declare that the research was conducted in the absence of any commercial or financial relationships that could be construed as a potential conflict of interest.

## References

[B1] Anter E. Duytschaever M. Shen C. Strisciuglio T. Leshem E. Contreras-Valdes F. M. (2018a). Activation Mapping With Integration of Vector and Velocity Information Improves the Ability to Identify the Mechanism and Location of Complex Scar-Related Atrial Tachycardias. *Circulation: Arrhythmia and Electrophysiology* 11 1–11. 10.1161/Circep.118.006536 30354312

[B2] Anter E. Kleber A. G. Rottmann M. Leshem E. Barkagan M. Tschabrunn C. M. (2018b). Infarct-Related Ventricular Tachycardia: Redefining the Electrophysiological Substrate of the Isthmus During Sinus Rhythm. *JACC Clin Electrophysiol* 4 1033–1048. 10.1016/j.jacep.2018.04.007 30139485

[B3] Anter E. Tschabrunn C. M. Buxton A. E. Josephson M. E. (2016). High-Resolution Mapping of Postinfarction Reentrant Ventricular TachycardiaClinical Perspective. *Circulation* 134 314–327. 10.1161/CIRCULATIONAHA.116.021955 27440005 PMC5072375

[B4] Brunckhorst C. (2002). Ventricular mapping during atrial and ventricular pacing. Relationship of multipotential electrograms to ventricular tachycardia reentry circuits after myocardial infarction. *European Heart Journal* 23 1131–1138. 10.1053/euhj.2001.3110 12090752

[B5] Cantwell C. D. Roney C. H. Ng F. S. Siggers J. H. Sherwin S. J. Peters N. S. (2015). Techniques for automated local activation time annotation and conduction velocity estimation in cardiac mapping. *Computers in Biology and Medicine* 65 229–242. 10.1016/j.compbiomed.2015.04.027 25978869 PMC4593301

[B6] Dubois R. Labarthe S. Coudiere Y. Hocini M. Haissaguerre M. (2012). Global and directional activation maps for cardiac mapping in electrophysiology. *Computing in Cardiology* 39 349–352.

[B7] Fitzgerald T. N. Rhee E. K. Brooks D. H. Triedman J. K. (2003). Estimation of Cardiac Conduction Velocities Using Small Data Sets. *Annals of biomedical engineering* 31 250–261. 10.1114/1.1543936 ^∗^year, 12680723

[B8] Gaeta S. Bahnson T. D. Henriquez C. (2020). Mechanism and magnitude of bipolar electrogram directional sensitivity: Characterizing underlying determinants of bipolar amplitude. *Heart Rhythm* 17 777–785. 10.1016/j.hrthm.2019.12.010 31843674 PMC8183728

[B9] Gokhale T. A. Asfour H. Verma S. Bursac N. Henriquez C. S. (2018). Microheterogeneity-induced conduction slowing and wavefront collisions govern macroscopic conduction behavior: A computational and experimental study. *PLoS Comput Biol* 14:e1006276. 10.1371/journal.pcbi.1006276 30011279 PMC6062105

[B10] Irie T. Yu R. Bradfield J. S. Circulation M. V. (2015). Relationship between sinus rhythm late activation zones and critical sites for scar-related ventricular tachycardia: A systematic analysis of isochronal late activation mapping. *Circ. Arrhythm. Electrophysiol.* 8 390–399. 10.1161/CIRCEP.114.002637 25740836 PMC4695215

[B11] Jackson N. Gizurarson S. Viswanathan K. King B. Masse S. Kusha M. (2015). Decrement Evoked Potential Mapping: Basis of a Mechanistic Strategy for Ventricular Tachycardia Ablation. *Circulation: Arrhythmia and Electrophysiology* 8 1433–1442. 10.1161/CIRCEP.115.003083 26480929

[B12] Jaïs P. Maury P. Khairy P. Sacher F. Nault I. Komatsu Y. (2012). Elimination of Local Abnormal Ventricular Activities: A New End Point for Substrate Modification in Patients with Scar-Related Ventricular Tachycardia. *Circulation* 125 2184–2196. 10.1161/CIRCULATIONAHA.111.043216 22492578

[B13] Josephson M. E. Anter E. (2015). Substrate Mapping for Ventricular Tachycardia: Assumptions and Misconceptions. *JACC Clin Electrophysiol* 1 341–352. 10.1016/j.jacep.2015.09.001 29759461

[B14] King J. H. Huang C. L.-H. Fraser J. A. (2013). Determinants of myocardial conduction velocity: implications for arrhythmogenesis. *Front Physiol* 4:154. 10.3389/fphys.2013.00154 23825462 PMC3695374

[B15] Leshem E. Tschabrunn C. M. Jang J. Whitaker J. Zilberman I. Beeckler C. (2017). High-Resolution Mapping of Ventricular Scar: Evaluation of a Novel Integrated Multielectrode Mapping and Ablation Catheter. *JACC Clin Electrophysiol* 3 220–231. 10.1016/j.jacep.2016.12.016 29759516

[B16] Masse S. Magtibay K. Jackson N. Asta J. Kusha M. Zhang B. (2016). Resolving Myocardial Activation With Novel Omnipolar Electrograms. *Circulation: Arrhythmia and Electrophysiology* 9 e004107. 10.1161/CIRCEP.116.004107 27406608 PMC4956680

[B17] Mendonca Costa C. Anderson G. C. Meijborg V. M. F. O’Shea C. Shattock M. J. Kirchhof P. (2020). The Amplitude-Normalized Area of a Bipolar Electrogram as a Measure of Local Conduction Delay in the Heart. *Front Physiol* 11:465. 10.3389/fphys.2020.00465 32508676 PMC7248250

[B18] Nayyar S. Wilson L. Ganesan A. N. Sullivan T. Kuklik P. Chapman D. (2014). High-density mapping of ventricular scar: a comparison of ventricular tachycardia (VT) supporting channels with channels that do not support VT. *Circulation: Arrhythmia and Electrophysiology* 7 90–98. 10.1161/CIRCEP.113.000882 24382409

[B19] Plank G. Hofer E. (2018). Use of Cardiac Electric Near-Field Measurements to Determine Activation Times. *Annals of biomedical engineering* 31 1066–1076. 10.1114/1.1603258 14582609

[B20] Schuler S. Keller M. W. Oesterlein T. Seemann G. Dossel O. (2013). Influence of Catheter Orientation, Tissue Thickness and Conduction Velocity on the Intracardiac Electrogram. *Biomedizinische Technik/Biomedical Engineering* 58 1–2. 10.1515/bmt-2013-4334 24043070

[B21] Shors S. M. Sahakian A. V. Sih H. J. Swiryn S. (1996). A method for determining high-resolution activation time delays in unipolar cardiac mapping. *Computing in Cardiology* 43 1192–1196. 10.1109/10.544343 9214838

[B22] Spach M. S. Barr R. C. Serwer G. A. Kootsey J. M. Johnson E. A. (1972). Extracellular potentials related to intracellular action potentials in the dog Purkinje system. *Circ Res* 30 505–519. 5026754 10.1161/01.res.30.5.505

[B23] Sulkin M. S. Laughner J. I. Hilbert S. Kapa S. Kosiuk J. Younan P. (2018). Novel Measure of Local Impedance Predicts Catheter–Tissue Contact and Lesion Formation. *Circulation: Arrhythmia and Electrophysiology* 11 e005831. 10.1161/CIRCEP.117.005831 29618475

[B24] Verma B. Loewe A. Luik A. Schmitt C. Doessel O. (2016). “Regional Conduction Velocity Calculation based on Local Activation Times: A Simulation Study on Clinical Geometries,” in *Proceeding of the 43rd Computing in Cardiology Conference*, (Vancouver, CA: Institute of Electrical and Electronics Engineers (IEEE)), 10.22489/cinc.2016.285-253

[B25] Verma B. Oesterlein T. Loewe A. Luik A. Schmitt C. Dössel O. (2018). Regional conduction velocity calculation from clinical multichannel electrograms in human atria. *Computers in Biology and Medicine* 92 188–196. 10.1016/j.compbiomed.2017.11.017 29223114

[B26] Zheng Y. Xia Y. Carlson J. Kongstad O. Yuan S. (2017). Atrial average conduction velocity in patients with and without paroxysmal atrial fibrillation. *Clinical Physiology and Functional Imaging* 37 596–601. 10.1111/cpf.12342 26762841

