# Supplementary material for: High-Resolution Measurement of Local Activation Time Differences From Bipolar Electrogram Amplitude
Source: Front Physiol. 2021 Apr 22;12:653645. doi: 10.3389/fphys.2021.653645 (PMC8100452; doi:10.3389/fphys.2021.653645)
Supplement: Supplementary file 1 [file Data_Sheet_1.docx]

# Supplementary Material

## Patient study

10 patients with a history of AF (5 persistent, 5 paroxysmal) undergoing a planned ablation procedure were enrolled. Patients were excluded if they had undergone prior left atrial ablation (including ablation of fractionated electrograms, roof line, ﬂoor line or posterior wall isolation) apart from pulmonary vein isolation (PVI). The patients had mean age of 68.4 years (range 57-78) and for all but one patient this was a ﬁrst-time ablation. The remaining patient had undergone a PVI procedure at our institution.

## Clinical data collection

High density maps of the left atrial posterior wall (LAPW) were created using the Carto3 electroanatomic mapping system (Biosense Webster, Diamond Bar, CA). Data were collected following PVI, after conﬁrming pulmonary vein entrance and exit block, and prior to any extra-pulmonary vein ablation. Patients who presented in AF were ﬁrst electrically cardioverted to sinus rhythm. A detailed left atrial geometry was collected by fast anatomical mapping (FAM) with a multipolar catheter consisting of 20 electrodes distributed on ﬁve separate splines (2-6-2 PentaRay catheter, Biosense Webster). Pacing was performed from the mid-coronary sinus at 600 ms cycle length with output 5 mA and pulse duration 2 ms. There were no complications or arrhythmias induced by this pacing protocol.

Electrograms were recorded point-by-point using a PentaRay multipolar mapping catheter and points acquired outside of the LAPW were discarded. Unipolar electrograms were recorded relative to Wilson’s central terminus and bandpass ﬁltered at 2-240Hz prior to export. Signals were sampled at the nominal rate of 1kHz with 0.003mV voltage steps.

Following the clinical procedure, data were de-identiﬁed and exported using the built-in Carto export utilities. Patient data sets were exported from the Carto system and all subsequent analyses were performed oﬄine using a custom software platform written in the Matlab programming environment (Mathworks Inc). Points were automatically excluded from further analysis if they were recorded more than 7mm from the collected geometry. Prior to bipole calculation the unipolar electrograms were additionally band pass ﬁltered from 5 to 100Hz.

## Computational Model

The computational tissue was modeled using the monodomain formulation given by

$$\begin{aligned} \nabla\cdot\left( \sigma_{i}\nabla V_{m} \right)=\beta\left( C_{m}\frac{\partial V}{\partial t}+I_{ion}\left( \boldsymbol{q},V_{m} \right)+I_{stim} \right), \end{aligned}\left( 3 \right)$$

where *σ_i_* is the intracellular conductivity tensor, *V_m_* is the transmembrane potential, *β* is the membrane surface area to volume ratio, *C_m_* is the speciﬁc membrane capacitance, *I_ion_* is the total ionic current which is function of several state variables **q** and the transmembrane potential and *I_stim_* is the intracellular stimulus. The membrane currents *I_ion_* were based on the Courtemanche *et al.* model (Courtemanche et al., 1998).

Unipolar electrograms were computed at sites along a line in the direction of propagation. The monolayer tissue was assumed to be immersed in an unbounded isotropic volume conductor. Following the approach used by Jacquemet and Henriquez, the electrical activity in this medium is generated by membrane current sources *I_m_* located in the plane of the monolayer (assuming the cells are tightly packed laterally). Using 2D monopolar source formulation, the electrode potential that is measured at point (*x, y*) is computed as

$$\begin{aligned} \Phi=\sum I_{m}Z, \end{aligned}\left( 4 \right)$$

where for a point electrode

$$\begin{aligned} Z=\frac{1}{4\pi\sigma_{e}\sqrt{(r^{2}+z^{2})}}, \end{aligned}\left( 5 \right)$$

where *r* is distance between the source and the electrode location in the plane of the tissue and *z* is the height of the electrode above the tissue. Electrodes with non-negligible size can be simulated by modifying the transfer function *Z*. For the case of a disc electrode of diameter *d_e_*, the transfer function is given by

$$\begin{aligned} Z=\frac{1}{2\pi\sigma_{e}d_{e}}\left( \frac{d_{e}}{\sqrt{\left( r-d_{e}/2 \right)^{2}+z^{2}}+\sqrt{\left( r+d_{e}/2 \right)^{2}+z^{2}}} \right). \end{aligned}\left( 6 \right)$$

When the disc diameter tends to 0, the formula is consistent with Eq.5. 21 simulated disc electrodes with a diameter of 200*μm* were positioned with an interelectrode distance of 0.5 mm and a distance of 1.5 mm above the monolayer near the center of the 3 cm long domain to minimize end eﬀects. Note that the height was chosen to better match the peak to trough timing of clinical unipolar electrograms. The conduction velocity was modiﬁed by assigning a linear change in intracellular conductivity from the default value of 4 *mS/cm* to 0.8 *mS/cm.*

The simulation code was written in MATLAB with the membrane model obtained from the CellML Model Respository (https://models.cellml.org/cellml). Equation 3 was solved using an explicit ﬁnite diﬀerence method with a *dt* = 0*.*005ms and spatial step sizes of *dx* = *dy* = 0*.*01 cm. To simulate fibrosis, nodes and connections along a line (i.e., the fiber direction) were deleted every 400 um. A node and its connections were deleted if a uniform random number between 0 to 1 was less than a user defined fraction. To enable stimulation along any edge, no boundary nodes were deleted. Once the nodes were deleted, the associated finite difference matrix was reconstructed to reflect these internal discontinuities by applying appropriate no flux conditions at the missing nodes.

## Cross-correlation time delay

For each clinical electrode pair we additionally calculated the time-delay between unipolar EGMs using cross-correlation, an established digital signal processing technique for measuring small time diﬀerences between signals[17, 7]. Prior to this analysis, both unipolar electrograms in a given pair were upsampled to 10,000 Hz using a cubic spline. Unipolar recordings were windowed to a period including the time of maximum negative *dV/dt* for both unipolar signals *±*15ms. To isolate the unipolar downstrokes, all time points with *dV/dt >* 0 were set to 0 mV. Cross correlation was performed using the default MATLAB implementation and the LAT diﬀerence was calculated as the timing of the maximum of the resulting correlation function.

# Mathematical model derivation

We modeled the unipolar electrogram as a traveling sine wave in one dimension, and a pair of unipolar electrograms (*V*_1_ and *V*_2_) as simultaneous measurements at sites separated by distance *d* (the inter-electrode spacing; Fig. 2A), where

$$\begin{aligned} V_{1}=A\sin\left( kx-\omega t \right) \end{aligned}\left( 7 \right)$$

$\begin{aligned} V_{2}=A\sin\left( k\left( x-d \right)-\omega t \right), \end{aligned}\left( 8 \right)$

and *A* is half of the peak to peak amplitude of the unipolar electrogram $(A=U/2)$. The bipolar electrogram, *V_B_*, is a superposition of these two unipolar waves,

$$\begin{aligned} V_{B}=V_{1}+\left( -V_{2} \right). \end{aligned}\left( 9 \right)$$

We sought to precisely determine the phase diﬀerence acquired during travel from one site to the next, which could then be related to the diﬀerence in local activation times (inter-electrode time delay) at these two locations. By modeling the electrogram as a periodic wave, the bipolar voltage amplitude can be quantitatively related to the phase diﬀerence between its component unipolar signals. The constraints of this assumption are discussed below. Importantly, we hypothesized that this methodology would allow small diﬀerences in phase to be measured by larger, measurable diﬀerences in amplitude. This concept is akin to interferometry methods in other physical systems, in which wave interference allows measurements of distance or time to be made in the amplitude/intensity domain.

Deﬁning the phase diﬀerence between the two waveforms as *φ* = *−kd*, the model becomes

$$\begin{aligned} V_{1}=A\sin\left( kx-\omega t \right) \end{aligned}\left( 10 \right)$$

$\begin{aligned} V_{2}=A\sin\left( kx-\omega t+\phi\right), \end{aligned}\left( 11 \right)$

Deﬁning the bipolar electrogram as above (Eq. 9), its amplitude, *B*, can be found by phasor subtraction

of Equations 10 and 11 to be

$$\begin{aligned} B=2A\sin\frac{\phi}{2} \end{aligned}\left( 12 \right)$$

Or

$$\begin{aligned} B=U\sin\frac{\phi}{2} \end{aligned}\left( 13 \right)$$

In this model, the peak bipolar amplitude is therefore related to the phase diﬀerence between its component unipolar signals by Eq. 13. As seen in Fig. S2A, this predicts a monotonic, 1:1 relationship within the range *−*180*^◦^ < φ <* 180*^◦^*, suggesting that the underlying unipolar phase diﬀerence can be accurately derived from the peak bipolar amplitude by

$$\begin{aligned} \begin{aligned} \phi=2\sin^{-1} \frac{B}{U}, \end{aligned}\left( 14 \right) \end{aligned}$$

Note that if a small angle approximation is made (where *B/U* is small), this becomes

$$\begin{aligned} \begin{aligned} \phi=\frac{2B}{U} \end{aligned}\left( 15 \right) \end{aligned}$$

This phase diﬀerence can be transformed into a time delay as follows. Substituting *φ* = *−kd* back into Eq. 14 gives an equation to relate the bipolar amplitude to the spatial frequency (or wavenumber), *k*, as

$$\begin{aligned} \begin{aligned} k=\frac{-2}{d}\sin^{-1} \frac{B}{U}. \end{aligned}\left( 16 \right) \end{aligned}$$

Knowing that the spatial frequency is related to the phase velocity by

$$\begin{aligned} v_{p}=\frac{\omega}{k}, \end{aligned}\left( 17 \right)$$

and

$$\begin{aligned} v_{p}=\frac{d}{\tau}, \end{aligned}\left( 18 \right)$$

where *τ* is the time lag between signals, *k* can be deﬁned as

$$\begin{aligned} k=\frac{\tau\omega}{d}. \end{aligned}\left( 19 \right)$$

Equation 16 becomes

$$\begin{aligned} \begin{aligned} \tau=\frac{-2}{\omega}\sin^{-1} \frac{B}{U}. \end{aligned}\left( 20 \right) \end{aligned}$$

This derivation demonstrates that in this simpliﬁed model, if two unipolar electrograms have equal amplitude and frequency, the peak of their resulting bipolar electrogram can be used to calculate a time delay between the two signals (Eq. 20).

To ﬁnd an activation time delay, the frequency of the unipolar downstrokes (*ω*) must be known. The frequency of each unipolar signal can be approximated using its maximum negative *dV/dt*, as follows. Given a sinusoidal approximation, the angular frequency can be found by equating a clinically measured maximum negative *dV/dt* (*m*) with the maximum slope of a sinusoid. The slope of the sinusoidal approximation is

$$\begin{aligned} \frac{\partial V}{\partial t}=-A\cos(kx-\omega t) \end{aligned}\left( 21 \right)$$

with maximum negative slope, *m*, therefore

$$\begin{aligned} m=-A\omega\end{aligned}\left( 22 \right)$$

In our model, therefore,

$$\begin{aligned} \omega=\frac{-2m}{U}. \end{aligned}\left( 23 \right)$$

The period of the downstroke, *T* , noting the standard relationship $T=2\pi/\omega$, is therefore

$$\begin{aligned} T=-\pi\frac{U}{m}. \end{aligned}\left( 24 \right)$$

Using Equation 20, this results in the following equation to calculate a time lag between (amplitude normalized) unipolar signals using only their bipolar amplitude and their maximum negative *dV/dt*,

$$\begin{aligned} \tau=\frac{U}{m}\sin^{-1} \frac{B}{U}. \end{aligned}\left( 25 \right)$$

At small phase diﬀerences, this can be further simpliﬁed to

$$\begin{aligned} \tau=\frac{B}{m}. \end{aligned}\left( 26 \right)$$

## Model constraints

To demonstrate the validity of using the bipolar peak amplitude to calculate unipolar time delays, it is necessary to demonstrate that (1) this relationship is monotonic and 1 to 1, (2) this relationship holds regardless of the order of unipolar activation sequence, and (3) the bipolar peak will always occur during a period of the electrogram when model assumptions are valid.

1. As seen in Equation 13 and Figure S2A, this analysis predicts a monotonically increasing bipolar amplitude from nadir to peak as unipolar phase diﬀerence changes from -180*^◦^* to +180*^◦^*. In the case of a unipolar amplitude, *U* = 1, the peaks will occur at -1 and +1, as shown. Outside of this range, the relationship between phase change and bipolar amplitude is no longer 1 to 1 and this approach is therefore limited to phase diﬀerences within *±*180*^◦^*.
2. Because it is not known *a priori* which unipolar in a given electrode pair will lead the other, this derivation must apply regardless of unipolar activation sequence. A change in time lag from positive (*τ >* 0, with unipolar 2 leading) to negative (*τ <* 0, with unipolar 1 leading) will change the bipolar peak overlying the unipolar downstrokes from a positive to a negative maximum (Fig. S1, left). Given equal positive and negative peak amplitudes in a sinusoid, however, the derived relationship between bipolar peak amplitude (*B*) and unipolar time delay (*τ* ) applies to either a positive or negative bipolar peak and is therefore independent of unipolar activation sequence.
3. This model is an approximation of the unipolar downstroke only and therefore is only valid during the period when both unipolar signals are in their downstroke. The bipolar peak must therefore occur prior to the completion of either unipolar signal’s downstroke to be safely interpreted according to the predictions of the simpliﬁed model. The peak of the bipolar electrogram will always occur within this period, as shown below.

### Timing of the bipolar peak

Given the dependence of bipolar peak amplitude on *dV/dt* (*m* in Eq. 25), for a given unipolar time delay, the bipolar waveform will always have greatest amplitude during the period of maximum unipolar slope (the downstroke). To ensure that the peak occurs within the period of simultaneous unipolar downstrokes, however, we must determine the phase of the peaks of the bipolar sinusoid relative to the unipolar downstrokes.

Given that in our model the unipolar signals have equal amplitude and frequency, the bipolar electrogram will have this same frequency but distinct amplitude and phase. The phase of the bipolar signal will be delayed relative to the ﬁrst unipolar signal (*U*_1_) by a phase delay of *φ_B_*. By phasor subtraction, *φ_B_* (expressed relative to *U*_1_) is

$$\begin{aligned} \phi_{B}=\tan^{-1} \frac{-\sin\phi}{1-\cos\phi}. \end{aligned}\left( 27 \right)$$

As diagrammed in Fig. S1A, in the case of leftward shifts of unipolar 2 (*φ >* 0), the relevant peak bipolar amplitude will be positive (*V*_1_ *− V*_2_ *>* 0) and the phase of its peak (*φ_p_*) will be the same as the overall bipolar signal (*φ_p_* = *φ_B_*).

As diagrammed in Fig. S1B, in the case of rightward shifts of unipolar 2 (*φ <* 0), the relevant peak bipolar amplitude will be a negative value (*V*_1_ *− V*_2_ *<* 0). Because the relevant peak will be its nadir, this peak will occur with an additional *−*180*^◦^* phase angle relative to the overall bipolar signal (*φ_p_* = *φ_B_ −* 180*^◦^*).

The phase angle of the bipolar peak (*φ_p_*) relative to unipolar 1 is therefore

$$\begin{aligned} \phi_{p}=\left\{ \begin{aligned} \phi_{B}-180^{\circ}, \phi<0^{\circ} \\ \phi_{B,}, \phi>0^{\circ} \end{aligned} \right. \end{aligned}\left( 28 \right)$$

This relationship is seen in Fig. S2B (black line).

As diagrammed in Fig. S1 (right), the phase of simultaneous downstrokes, *θ*, is

$$\begin{aligned} 180^{\circ}<\theta<\phi, for \phi<0^{\circ} \end{aligned}\left( 29 \right)$$

$$\begin{aligned} \phi-180^{\circ}<\theta<0^{\circ}, for>0^{\circ}. \end{aligned}\left( 30 \right)$$

As seen in Fig. S2B, the bipolar peak will therefore always occur within the phase of simultaneous unipolar downstrokes (shaded area). Note that this ﬁnding is unchanged whether the unipolar is modeled as a cosine wave (with peak at phase 0*^◦^*) or a sine wave (in which the above relationships would all be displaced by 90*^◦^*).

**Fibrosis model**

To explore the performance of the DELTA method on more complex EGMs, additional 2D simulations were performed incorporating simulated fibrosis. Fibrosis was modeled by random deletion of 35% of discretized elements (sized dx*dy = 1e-4 cm^2^) within the 2x2cm domain thereby creating areas of discontinuous activation. Simulated EGMs were created (as above) from a 5x5 grid of disc electrodes with diameter 400um located 1.5mm above the tissue. Electrodes were separated by 1mm center-to-center and bipolar EGMs calculated from all pairs of adjacent electrodes during activation by a plane wave initiated from the inferior border of the domain. To simulate clinical data, unipolar EGMs were filtered and down-sampled to 1kHz prior to calculation of their resulting bipolar EGM.

An example of fractionated EGMs is seen in Fig. S6. In the presence of fibrosis, local unipolar EGMs (red and blue) and the resulting calculated bipolar EGM (black) show a complex morphology with notching and multiple peaks (Fig. S6B). The raw EGMs (i) and the 1kHz, filtered EGMs are shown (ii). For this electrode pair, the ground truth LAT difference of -1.665ms was determined by threshold crossing of the membrane voltage (V_m_) at each site (Fig. S6A). The LAT difference measured by the standard method (maximum negative dV/dt timing) was -2.730ms and -3ms for the raw and down-sampled EGMs, respectively. The LAT difference measured by the DELTA method was -1.036ms and -1.552ms for the raw and down-sampled EGMs, respectively.

A total of 20 unique models were run, from which 243 bipolar EGMs were found with small time delays (LAT difference <= 2ms calculated by Vm threshold crossing). LAT differences for each electrode pair were found by ground truth Vm threshold crossing, the standard method (timing of maximum negative dV/dt), and the DELTA method. Compared to the ground truth, the mean errors in LAT were 3.112ms (standard deviation 3.776ms) and 1.1776ms (standard deviation 0.904ms) for the standard method and the DELTA method, respectively.

# Supplemental ﬁgure legends

**Figure S1. Defining the phase of the bipolar peak and simultaneous unipolar down- strokes.** See supplemental text for full description.

**Figure S2. The peak bipolar amplitude encodes the unipolar phase diﬀerence and will occur during the period of simultaneous unipolar downstrokes.** (A) The peak bipolar amplitude, *B*, is predicted to vary as a function of the phase diﬀerence, *φ*, between its component unipolar electrograms by Eq. 13. This model predicts that the bipolar amplitude will have a 1:1, monotonic relationship with unipolar phase diﬀerence within the range of *−*180*^◦^ < φ <* 180*^◦^* (dashed lines). The curve shown is calculated for the case of 1mV unipolar amplitude. (B) The phase angle of the bipolar peak (*φ_p_*) will always occur during the period of simultaneous unipolar downstrokes (shaded area), during which period model assumptions are valid.

**Figure S3. Characteristics of clinically recorded unipolar signals.** (A) Histogram of peak-to-peak unipolar amplitudes. (B) The maximum negative *dV/dt* measured for each unipolar electrogram is linearly correlated with its unipolar amplitude. Linear regression is shown in red. (C) Histogram of relative amplitude diﬀerences between electrodes in bipolar pairs. (D) Comparison of each bipolar EGM’s absolute peak amplitude with the mean amplitude of it’s component unipolar signals. Data in C and D includes all bipolar pairs (all inter-electrode spacings).

**Figure S4. Distribution of maximum negative** *dV/dt* **in amplitude normalized unipolar electrograms.** After normalizing unipolar amplitudes to 0 to 1mV, measured *dV/dt* ranges from approximately -0.3 to -0.05mV/ms.

**Figure S5. Inter-electrode time delays calculated by DELTA correlate with time domain methods.** The correlation between mean calculated time delays and the standard measurement of activation time diﬀerence (by maximum negative *dV/dt* timing, black) compares favorably to time delays measured by cross-correlation (cyan).

**Figure S6. Example LAT difference calculation in complex EGMs.**  (A) Ground truth LATs (dashed lines) were found by threshold crossing of Vm at each electrode’s location. (B) Simulated unipolar EGMs at each site (red and blue) were used to calculate a bipolar EGM (black). Due to discontinuous activation, multiple peaks and notching were seen in the resulting EGMs. LATs were determined by the maximum negative dV/dt timing (dashed lines) and used to calculate a standard LAT difference. (C) The amplitude-normalized unipolar EGMs (red and blue) and the peak amplitude of their resulting calculated bipolar EGM (black) were then used to calculate a LAT difference by the DELTA method. Shown are the raw EGMs (i) and the more clinically realistic 1kHz down-sampled and filtered EGMs (ii). See supplement text for details.


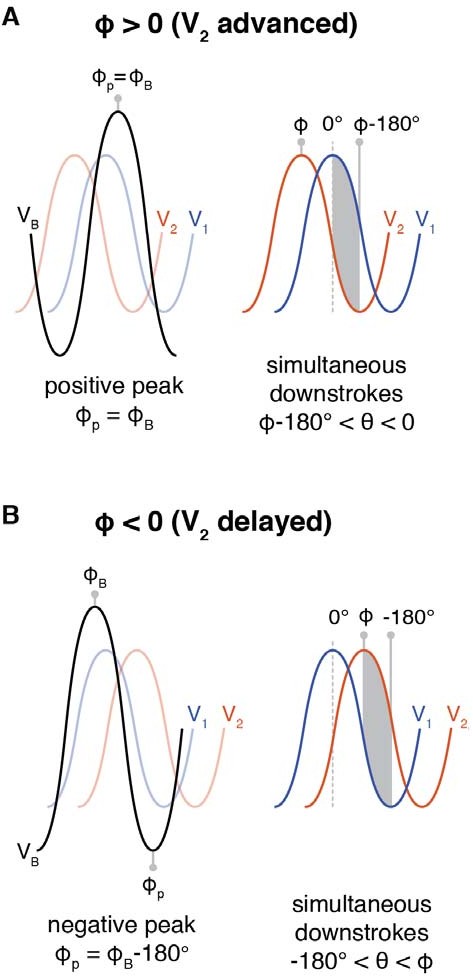


Figure S1:


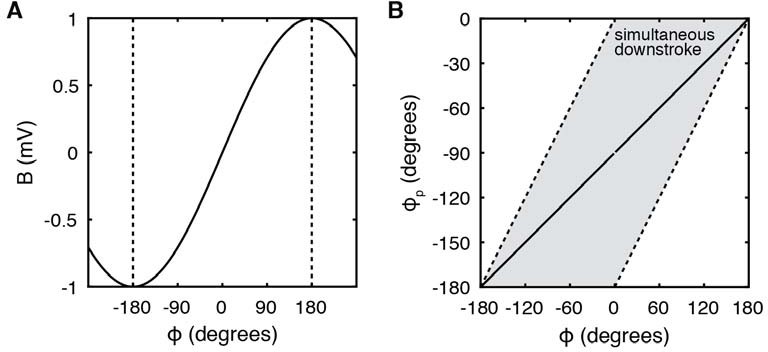


Figure S2:


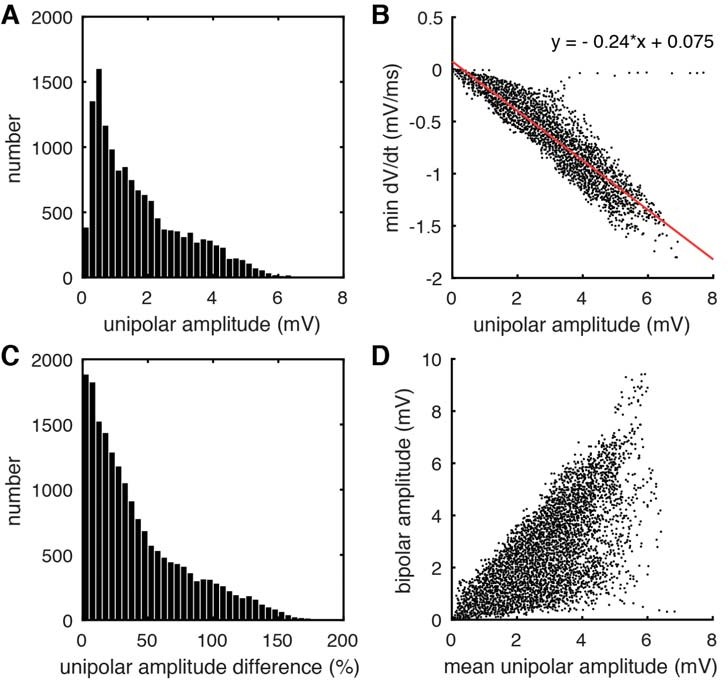


Figure S3:


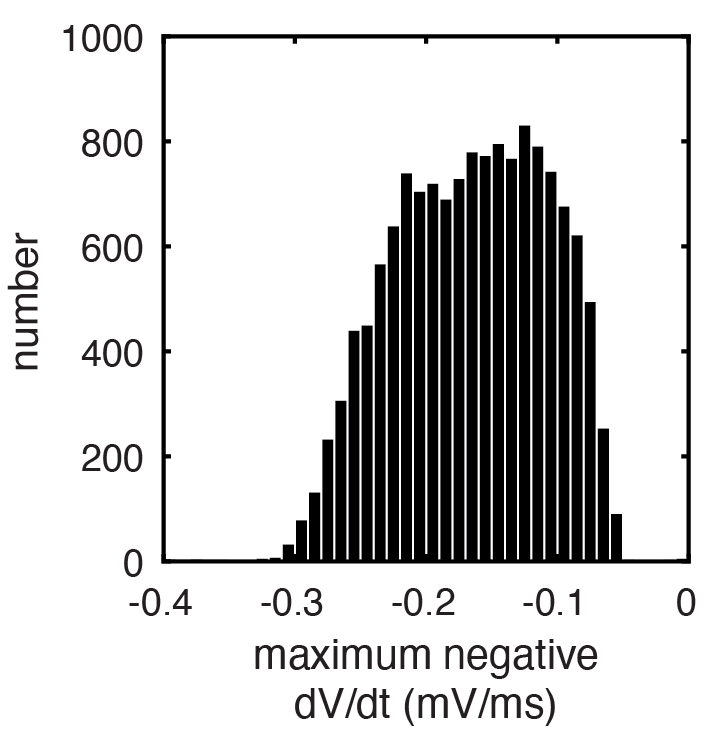


Figure S4:


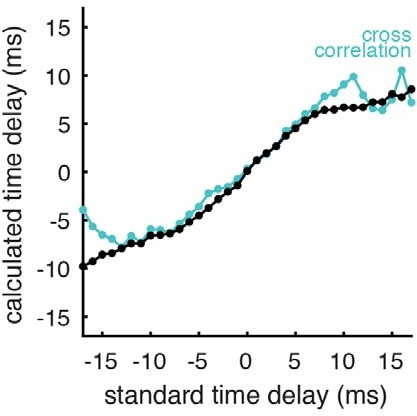


Figure S5:


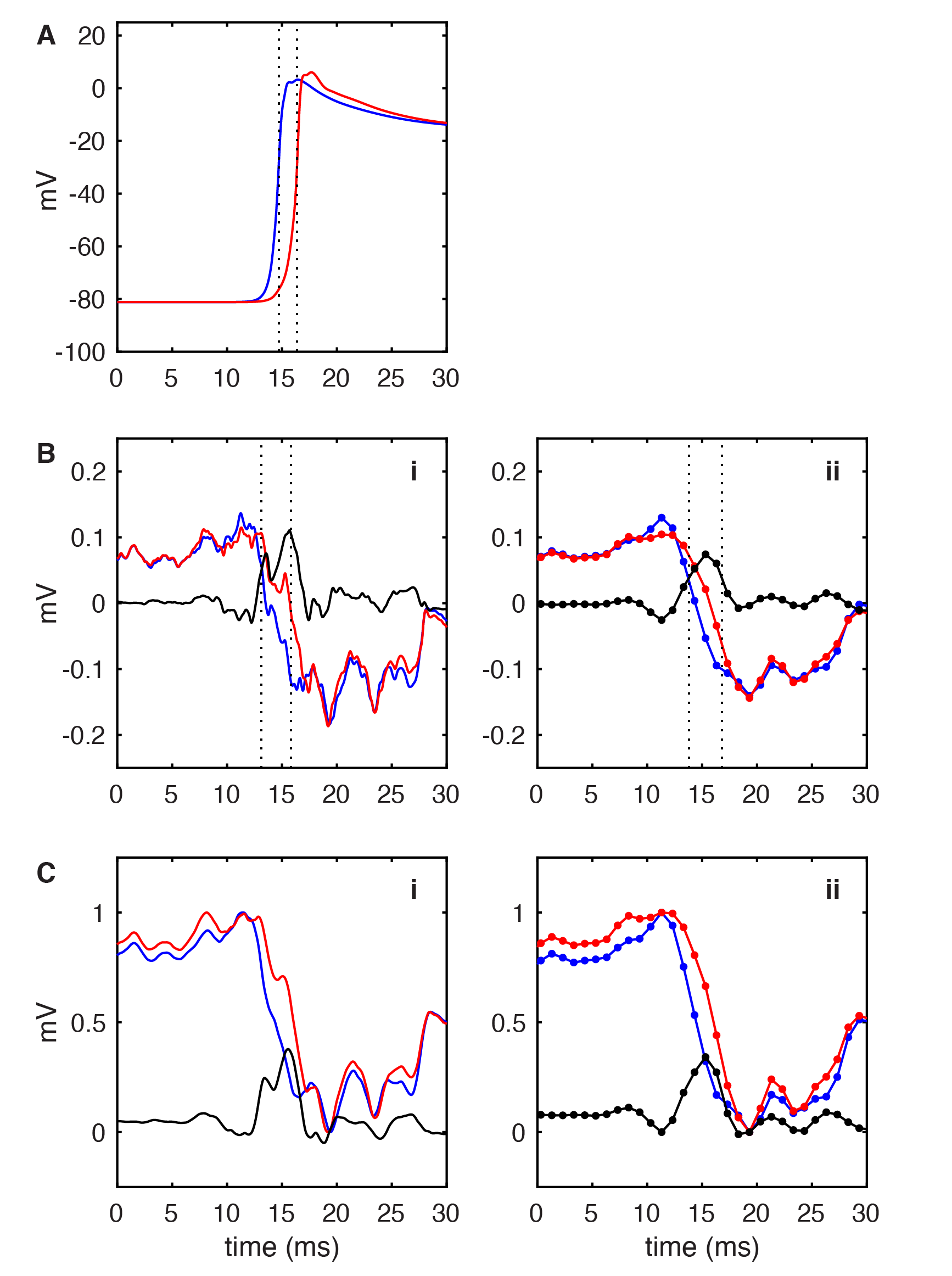


Fig S6

**REFERENCES**

Courtemanche, M., Ramirez, R. J., and Nattel, S. (1998). Ionic mechanisms underlying human atrial action potential properties: insights from a mathematical model. *Am J Physiol* 275, H301–21. doi:10.1152/ajpheart.1998.275.1.H301.
